# Investigating medical tourism development indicators in Yazd Reproductive Sciences Institute: A cross-sectional study

**DOI:** 10.18502/ijrm.v20i12.12565

**Published:** 2023-01-09

**Authors:** Milad Shafii, Farzan Madadizadeh, Roohollah Askari, Mohammad Zarezadeh, Ali Mohamad Abdoli, Sajjad Bahariniya

**Affiliations:** ^1^Department of Health Services Management, Health Policy and Management Research Center, School of Public Health, Shahid Sadoughi University of Medical Sciences, Yazd, Iran.; ^2^Center for Healthcare Data Modeling, Departments of Biostatistics and Epidemiology, School of Public Health, Shahid Sadoughi University of Medical Sciences, Yazd, Iran.; ^3^Research and Clinical Center for Infertility, Yazd Reproductive Sciences Institute, Shahid Sadoughi University of Medical Sciences, Yazd, Iran.

**Keywords:** Medical tourism, Infertility, Advertising, Marketing, Indicators, Reagents.

## Abstract

**Background:**

Payingspecial attention to the development indicators of medical tourism (MT) can lead to the further development of this industry and tourists' satisfaction.

**Objective:**

This study aimed to investigate MT development indicators in Yazd Reproductive Sciences Research Institute, Yazd, Iran in 2021.

**Materials and Methods:**

In this cross-sectional study, 430 medical tourists referred to the Yazd Reproductive Sciences Institute, Yazd, Iran in 2021 were studied. A researcher-made questionnaire with 46 questions and 10 dimensions was used in both internal and external environments to collect data. The Spearman correlation coefficient was also used to assess the correlation between the quantitative factors.

**Results:**

The reliability and validity of the scale were confirmed. The mean ± SD of the advertising and marketing index scored was lower than the other internal environmental indices (15.05 ± 2.16). Also, the economic factors and tourism infrastructure were lower than the other external environmental factors (9.8 ± 1.99, 8.53 ± 2.11, respectively).

**Conclusion:**

Given the importance of MT, top managers and relevant authorities should pay close attention to the criteria of advertising, marketing, and tourism infrastructure. MT demand can be improved by increasing the importance of advertising, implementing realistic advertising strategies, and developing adequate urban infrastructure and services.

## 1. Introduction

Medical tourism (MT) is growing at a breakneck pace and its potential can significantly contribute to nations' sustainable development and economic growth (1, 2). MT refers to the process by which individuals go to another city or nation to get nonemergency medical interventions that are unavailable, delayed, unprofessional, or too costly in their country of residence. According to applied research, the following factors contribute to individual motivations for engaging in MT: long waiting times, high costs, overcrowding, an insufficient number of healthcare professionals, and sophisticated technological equipment (3). The most common approaches of medical tourists are as follows: dental treatment, cosmetic surgery, orthopedic therapies, organ transplantation, and infertility treatment (4).

Investing in the MT sector is a strategy to increase gross domestic product, improve services, earn foreign currency, create a better trade balance, and improve the tourism industry (5). Health tourism is already a $100 billion business worldwide, and this part of tourism is currently enjoying an average increase of 25% (6). MT has a direct relationship with the health of medical tourists and is therefore very sensitive. Currently, many countries provide MT services only to earn money, while the nature of MT is different from other types of tourism and more potential is needed (7). Meeting the expectations of medical tourists and considering more benefits than treatment costs can lead to the success and development of MT (8).

Some studies have been conducted to assess the state of MT development indicators and to identify variables affecting the industry's growth (9, 10). The most critical dimensions of MT included: financial services, quality of medical services, political factors, information, attitudes of doctors and specialists in the field of healthcare and cultural factors (11). In a study, the factors affecting the attractiveness of MT in India were examined and it was concluded that the main reason for becoming a strong MT destination is because of its specialized and high-quality medical services at an affordable price. Researchers in this study judged service quality as a vital factor in MT that significantly affects patient satisfaction (12).

Although Yazd is a capable center in the field of infertility in Iran and across the world, there are no necessary tourism infrastructure services and facilities for tourists. Considering the importance of the above issue, this research was conducted to investigate the status of current MT development indicators at Yazd Reproductive Sciences Institute, Yazd, Iran.

## 2. Materials and Methods 

This cross-sectional study examined the state of MT development indicators at Yazd Reproductive Sciences Institute, Yazd, Iran in 2021 (for 9 months from February to November 2021). In this study, 430 internal medical tourists by convenience sampling were included. The most important criterion for entering this study was that tourists who refer to the infertility research center for treatment should be examined. Local people or non-tourists were excluded. In fact, only people outside of Yazd province were investigated and people from other cities of the province were not included in the study. In fact, only people outside of Yazd province were investigated and people from other cities of the province were not included in the study. If a person was originally from another city, but lived in Yazd for several years, that person was not considered a tourist. On the other hand, if a person is originally from Yazd but has lived in another city for several years, that person is considered a tourist. All participants were asked to fill a researcher-made questionnaire with 46 questions and 10 dimensions in both internal (factors inside the medical center) and external (factors outside the medical center) environments.

To make a questionnaire in the first phase, essential information was extracted from previous Persian and English studies (450 studies). These studies were extracted from various databases such as Scopus, PubMed, Science Direct, Web of Science, SID, Google Scholar, and others. The Delphi method was used to gather expert opinions. For this purpose, 3 phases of the Delphi method were used, so that in each phase, the importance of the variables was measured from the point of view of healthcare professionals, health policy makers, tourism managers, MT experts, and faculty members of Shahid Sadoughi University of Medical Sciences, Yazd. Face validity and content validity were evaluated. For content validity, the content validity ratio and content validity index were calculated (overall content validity index = 0.85). In construct validity questions were classified, and scale dimensions were determined through exploratory factor analysis. Then, the accuracy of this analysis was evaluated using confirmatory factor analysis. For validity assessment of the questionnaire, internal consistency and stability reliability were calculated. For internal consistency, Cronbach's alpha was 0.817 for this questionnaire. For stability reliability, the questionnaire's intra-cluster correlation coefficient was 0.779. The questionnaire contained 46 questions with 10 dimension { medical services (11 questions), human resources (6 questions), administrative and financial services (5 questions), support services (3 questions), information (3 questions), advertising and marketing (4 questions), economic factors (3 questions), cultural and political factors (5 questions), geographical and environmental factors (3 questions), and tourism industry infrastructure (3 questions) } . This 5-point Likert questionnaire included 5 response options (strongly agree, agree, have no opinion, disagree, and strongly disagree). The working method was that the scores of the questions of each item were added together and finally a comparison was made. The researcher asked the participants to complete the questionnaires.

### Sample size 

Using the Cochran prevalence sample size formula, by considering the prevalence of infertile women equal to 50%, and the significance level of 5%, an error rate of 5%, and the power test of 80%, the initial sample size was estimated to be 384, which considering a 10% chance of nonresponse rate, the final sample size was considered to be 430. The sample size was calculated based on a similar study (13).

### Ethical considerations

The proposal of the present study was approved by the Ethics Committee of Shahid Sadoughi University of Medical Sciences, Yazd, Iran (Code: IR.SSU.SPH.REC.1399.170). The informed consent form were obtained from all participants following the explanation about the goal of the study.

### Statistical analysis

Social package Sciences statistical v. 24 (SPSS, Inc, Chicago, IL) was used to perform all statistical analyses. Quantitative data were reported as mean ± standard deviation, median, minimum, maximum, and frequency (percentage). The Spearman tests were also used to assess the correlation between the quantitative factors. A p-value < 0.05 was considered statistically significant.

## 3. Results

Initially, 500 participants were eligible to enter the study. Out of them, 70 tourists were excluded due to lack of accuracy in completing the questionnaire. Finally, the data of 430 participants were analysed (Table I).

52.6% (n = 226) of participants were aged between 30-40 yr. The number of women was more than men (62.1%). 52.1% had the experience of a previous trip to Yazd. 40.7% knew this medical center through their doctor. The demographic characteristics of participants were shown in table I.

The descriptive statistics of MT development in the field of infertility based on the dimensions of the questionnaire was shown in table II.

The mean score of developmental indices in the internal environment was 139.21 ± 8.05, the mean total score of developmental indices in the external environment was 54.95 ± 4.37, and the mean score of the total questionnaire was 194.17 ± 10.45.

The descriptive statistics for MT development indicators were presented in table III. Our results indicated that medical services, human resources, administrative and financial services, support services, information, cultural and political factors, and geographical and environmental factors were all “very good."

The correlation between indicators of the internal and external environment was calculated using Spearman's correlation coefficient and showed that the correlation between these dimensions was significant and weak (r = 0.349, p < 0.001).

**Table 1:** The demographic characteristics of the study participants (n = 430)


**Variables**		**n (%)**
**Age (yr)**		
	**< 30**	144 (33.4)
	**30-40**	226 (52.6)
	**> 40**	60 (14.0)
**Gender**		
	**Male**	163 (37.9)
	**Female**	267 (62.1)
**Having travel experience to Yazd**		224 (52.1)
**Occupation**		
	**Housewife**	209 (48.6)
	**Self-employed**	127 (29.5)
	**Employee**	83 (19.3)
	**Hospital personnel**	11 (2.6)
**Education level**		
	**High school**	108 (25.1)
	**Diploma**	117 (27.2)
	**Associate degree**	71 (16.5)
	**B.Sc.**	112 (26.0)
	**Degree.Sc.**	17 (4.0)
	**Doctorate **	5 (2.1)
**Travel vehicle**		
	**Private car**	249 (57.9)
	**Bus**	73 (17.0)
	**Train**	57 (13.3)
	**Airplane**	51 (11.8)
**How to get acquainted with this medical center**		
	**Broadcasting**	43 (10.0)
	**Social media**	51 (11.9)
	**Website**	22 (5.1)
	**Acquaintances and relatives**	97 (22.6)
	**Friends**	42 (9.7)
	**Physician**	175 (40.7)
**Number of children**		
	**0**	314 (73.0)
	**1**	100 (23.3)
	**2**	16 (3.7)

**Table 2:** The mean of MT development indicators in the field of infertility by the dimensions of the questionnaire


**Dimensions**		**Items**	**Minimum**	**Maximum**	**Median**	**Mean ± SD**
**Internal environmental**						
	**Medical services**	1-11	11.00	55.00	48.00	47.91 ** ± ** 4.83
	**Manpower**	12-17	6.00	30.00	30.00	28.89 ** ± ** 1.94
	**Administrative and financial services**	18-22	5.00	25.00	22.00	21.46 ** ± ** 1.90
	**Support services**	23-25	3.00	15.00	13.00	12.76 ** ± ** 1.49
	**Information**	26-28	3.00	15.00	13.00	13.12 ** ± ** 1.76
	**Advertising and marketing**	29-32	4.00	20.00	15.00	15.05 ** ± ** 2.16
**External environmental**						
	**Economic factors**	33-35	3.00	15.00	10.00	9.89 ** ± ** 1.99
	**Cultural and political factors**	36-40	5.00	25.00	25.00	23.78 ** ± ** 1.64
	**Geographical and environmental** **factors**	41-43	3.00	15.00	13.00	12.74 ** ± ** 1.74
	**Tourism industry infrastructure**	44-46	3.00	15.00	9.00	8.53 ** ± ** 2.11
MT: Medical tourism						

**Table 3:** MT development indicators scoring


	**Score status (Ref)**					
**Dimensions**	**Very weak**	**Weak**	**Medium**	**Good**	**Very good**	Mean score*
	**Medical services**	< 11	11-22	22-33	33-44		44-55	47.91 ± 4.83
**Manpower**	< 6	6-12	12-18	18-24	24-30	28.89 ± 1.94
**Administrative and financial** **services**	< 5	5-10	10-15	15-20	20-25	21.46 ± 1.90
**Support services**	< 3	3-6	6-9	9-12	12-15	12.76 ± 1.49
**Information**	< 3	3-6	6-9	9-12	12-15	13.12 ± 1.76
**Advertising and marketing**	< 4	4-8	8-12	12-16	16-20	15.05 ± 2.16
**Economic factors**	< 3	3-6	6-9	9-12	12-15	9.89 ± 1.99
**Cultural and political factors**	< 5	5-10	10-15	15-20	20-25	23.78 ± 1.64
**Geographical and environmental** **factors**	< 3	3-6	6-9	9-12	12-15	12.74 ± 1.74
**Tourism industry infrastructure**	< 3	3-6	6-9	9-12	12-15	8.53 ± 2.11
**Internal environmental factors**	< 32	32-64	64-96	96-128	128-160	139.21 ± 8.05
**External environmental factors**	< 14	14-28	28-42	42-56	56-70	54.95 ± 4.37
*Data presented as Mean ± SD. MT: Medical tourism						

## 4. Discussion

Medical services were one of the key indicators investigated in the internal environment. The following medical services in this center were fully assessed: a short wait for services, the availability of high-quality specialized medical services, the presence of advanced medical facilities and equipment, comprehensive medical services, new medical techniques and methods, patient privacy, dignity and respect, the confidentiality of medical tourist information and records, and providing the essential para clinical services. In a study, researchers emphasized the important role of medical services in the growth of MT and the necessity of expanding high-tech medical services in Thailand (14). Also, other studies emphasized the importance of providing high-tech medical services as a factor in the growth of MT and increasing visitors' loyalty to MT (15, 16). The results of these studies confirm the findings of the present study on medical services. Human resource is another important factor for determining how the internal environment was evaluated in this center. Appropriate grading of human resources in this center can indicate the physician's high level of knowledge and skill, the physician's high level of credibility and international reputation, the physician's high level of professional responsibility and commitment, the presence of experienced and trained physicians and personnel, the physician's ability to communicate with medical tourists and the staff's appropriate behavior. A study showed that an effective workforce can be a critical factor in determining the growth of MT in Indonesia (17). In another study, researchers examined the policies promoting MT in South Korea, to develop human resource management guidelines for the MT industry based on the operational realities to ensure the availability of professional and trained human resources (18). These studies confirm the conclusions of the current study findings.

Administrative and financial services were another significant predictive factor of the internal environment. The situation of administrative and financial services of Yazd Reproductive Sciences Research Institute was evaluated as very good. This demonstrates the medical center's clarity and comprehensibility in all stages, the acceptability of tourism payment methods, the transparency of medical tariff rates for internal patients, and the tolerance of tourist medical visit costs. In a study, researchers emphasized the critical role of financial services in attracting medical tourists and argued that when healthcare costs increase, tourists face more financial risks and, ultimately, a lower rate of return. The number of visitors returning to the appropriate medical facilities is reduced (19). The findings of these studies confirm the current findings. The state of advertising and marketing in the domestic environment was evaluated as good. This might mean that the experienced personnel educates visitors about the facilities in this medical institution via face-to-face interactions, brochures or booklets, social media, and user-friendly websites. Similar studies by researchers, underlined the need for health authorities and managers to pay particular attention to the advertising and marketing index as an effective indicator of MT growth (20, 21) this research confirms the findings of the present study.

Economic, cultural, political, geographical, environmental factors, and tourism industry infrastructure were all investigated in the external environment. In the meantime, economic factors were assessed as good. This may indicate cost-effective travel to Yazd city, cost-effective accommodation, and recreation. Researchers also identified economic factors as an external factor affecting the growth of MT (22). One of the influential indicators in the external environment was the tourism industry infrastructure. This external index was evaluated as unfavorable compared to other indicators studied in the present study. It seems that the lack of a suitable website to meet the needs of health tourists, the lack of relaxing places to rest after receiving medical services, the lack of special centers to help health tourists, and the lack of adequate urban facilities are the most significant reasons for the inefficiency of this index. In a study, the major elements affecting the growth of MT in Turkey were evaluated and it was concluded that strategic marketing road maps should prioritize economic considerations and tourist infrastructure (23). The findings of this research confirm the findings current.

Managers of Yazd Reproductive Sciences Institute should pay close attention to the advertising and marketing index and make necessary plans. On the other hand, one of the ways to influence the attractiveness of MT and tourists' decisions to visit Yazd as a tourism destination is the tourism industry's infrastructure. Provincial senior managers should increase the demand for health tourism by enhancing the tourism industry's infrastructure index and adequate urban facilities.

##  Conflict of Interest

The authors declare that there is no conflict of interest.
